# A Grid Connected Photovoltaic Inverter with Battery-Supercapacitor Hybrid Energy Storage

**DOI:** 10.3390/s17081856

**Published:** 2017-08-11

**Authors:** Víctor Manuel Miñambres-Marcos, Miguel Ángel Guerrero-Martínez, Fermín Barrero-González, María Isabel Milanés-Montero

**Affiliations:** Power Electrical and Electronic Systems Research Group, Escuela de Ingenierías Industriales, Universidad de Extremadura, Avda. de Elvas, s/n, 06006 Badajoz, Spain; mguerrero@peandes.net (M.Á.G.-M.); fbarrero@unex.es (F.B.-G.); milanes@unex.es (M.I.M.-M.)

**Keywords:** photovoltaic power systems, inverters, energy storage, hybrid power systems, power generation planning

## Abstract

The power generation from renewable power sources is variable in nature, and may contain unacceptable fluctuations, which can be alleviated by using energy storage systems. However, the cost of batteries and their limited lifetime are serious disadvantages. To solve these problems, an improvement consisting in the collaborative association of batteries and supercapacitors has been studied. Nevertheless, these studies don’t address in detail the case of residential and large-scale photovoltaic systems. In this paper, a selected combined topology and a new control scheme are proposed to control the power sharing between batteries and supercapacitors. Also, a method for sizing the energy storage system together with the hybrid distribution based on the photovoltaic power curves is introduced. This innovative contribution not only reduces the stress levels on the battery, and hence increases its life span, but also provides constant power injection to the grid during a defined time interval. The proposed scheme is validated through detailed simulation and experimental tests.

## 1. Introduction

Energy Storage Systems (ESS) are urgently needed by the traditional electrical generation industry, which have almost no such storage capability. Traditional electricity transmission and distribution systems transport the electrical energy from large power plants to consumers in a unidirectional way. Due to this, electricity must be consumed by matching precisely the generation with demand, but the electricity demand fluctuates heavily and consequently power plants must be overdesigned which implies an inefficient and expensive electrical system [1].

Energy production could be made independent from the demand by means of ESS. With large-scale electricity storage capacity available, the generating capacity could be designed in electrical average terms rather than electrical peak terms. Subsequently, ESS could provide several advantages such as peaking power, standby reserve and load following. In addition, the harmful emissions of the thermal power sources could be reduced with the improved grid efficiency thanks to the fact ESS provide spinning reserves and dispatchable loads.

Furthermore, ESS is a needed technology for Distributed Energy Resource (DER) systems. A DER is meant to be as a sustainable and environmentally friendly alternative to the traditional energy system. DERs are usually installed in a distributed way, placed close to the consumers and designed on a scale of kW to MW, unlike conventional big power plants, which are installed in a centralized way. The energy system is evolving in a way the DERs have more presence, which implies the energy system is becoming a mix of centralized and distributed subsystems. As DER provide smaller capacity but a bettered suited to respond to drastic load fluctuations, ESS is the key in order to increase power flexibility and back up Uninterruptible Power Supplies (UPSs). The connection to the supply utility grid of combined RES-based generators and electric storage systems becomes a challenge [2].

DERs based on renewable energy systems such as solar photovoltaic (PV), wind turbines and wave, present an inherent intermittency and non-controllability, which depends on the weather. A well-sized ESS could deal with the energy generation unpredictability of this kind of DERs. Obviously, in periods of greater generation than demand the extra energy is stored, while in higher demand than generation periods the balance is taken from the stored energy [3].

### 1.1. Present State of the Art

**Figure 1:**
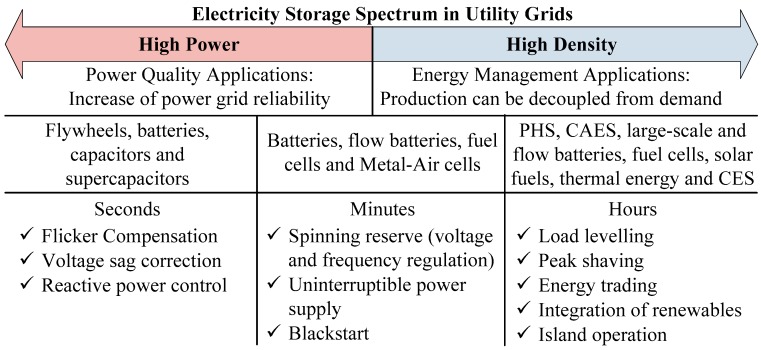
Energy storage systems technologies.

A meticulous techno-economic or cost-benefit analysis of ESS with consistent, updated cost data and a holistic cost analysis framework are required, in order to evaluate the life cycle costs of utility-scale electricity storage systems. Studies done in the past years, [4,5,6], including several energy storage technologies such as Pumped Hydropower Storage (PHS), Compressed Air Energy Storage (CAES), flywheel, electrochemical batteries, flow batteries, superconducting magnetic energy storage, supercapacitors (SCs), and hydrogen energy storage, reveals that batteries are a very interesting choice due to their energy density and recent drop in price. Although there are various commercially available ESS systems, no single storage system meets all the requirements for an ideal ESS (see Figure 1), that is, mature technology, long lifetime, low cost, high density, high efficiency and environmentally benign.

Battery versus Hybrid Energy Storage Systems (HESS) performance was studied in [7]. Passive, semi-active and fully active battery-supercapacitor hybridization improves battery only results when the load demands pulsed currents. On the one hand, passive hybrid is the simplest and cheapest solution but also carry some disadvantages, as it has no control. On the other hand, the fully active hybrids improve the performance, but the power stage needs two direct current/direct current (DC/DC) converters and the corresponding control circuitry.

One of the feasible solutions for photovoltaic renewable energy sources is hybridization of high-energy batteries with supercapacitors [8]. This energy source requires high power density plus high energy density. Modern battery technologies always lack one of them. Li-ion batteries present better power and energy density characteristics than others so they are the ones used for this kind of systems. Nevertheless, the power/energy trade-offs imply a non-optimized battery size as it provides the proper energy with excess of power and vice versa. Supercapacitor technology provides high power density and exhibits a rapid development at a reasonable cost. In addition, supercapacitors feature a better charge/discharge performance than any battery, involving a decrease in the system losses, which increases the system life because of the lower operating temperature.

### 1.2. Literature Review

Considering the promising battery-supercapacitor HESS combo, several published works [9,10,11,12,13,14,15,16,17,18,19,20,21,22,23,24,25,26,27] have been analyzed and are summarized in Table 1 and Table 2. From these selected documents, the following remarks can be extracted:

- Load supply is the most used application for low power. Another common application is energy harvesting. An interesting hybridization was done in [28,29] by means for water harvesting coupled to a disconnected system.
- Microgrids are the most used application for high power including energy management through global control with connection to grid when there is no energy stored. Some other applications are power injection regulation for tracking the forecasts, smoothing the fluctuating output power of the PV plant and power sharing.
- The trend when using ESS is to implement independent power stages for each power source, which means a boost stage for photovoltaics [30]. Photovoltaic inverters with two or more stages are usually implemented in the low-medium power range in order to boost the PV array voltage [31,32]. For higher power ranges, the boost stage is avoided as the PV arrays provide enough voltage and the efficiency is higher [33]. However, the energy management advantages introduced by an ESS require independent stages for each energy source in order to be optimized [34].
- Also, the control scheme is more flexible and robust when each decoupled stage introduces its degrees of freedom, to include all the electrical functions the converter is supposed to have. That is why most of the topologies include boost and DC/DC stages, including high power oriented ones, despite being worse. In addition, the efficiency of the boost stages could be optimized for the design [35].
- The main goal is usually to maintain the battery health, in addition to the particular application purpose. This is done using the supercapacitor for the sharp changes and developing control strategies that decrease the charge/discharge current rates of battery while maintaining a healthy state of charge (SOC) for both. These techniques also imply reduced current stress levels, less temperature and improved battery life span. Usually, the hybrid solution is done by using a low pass filter (LPF) which provides a high frequency reference for the supercapacitor and a low frequency reference for the battery.

### 1.3. Motivation and Objective

Photovoltaic panels are continuously improving and increasing their efficiency, but photovoltaics is still an intermittent energy generation form. A storage system is required to meet the load and function requirements of the applications. In general, lead acid batteries are used as a conventional storage technology for photovoltaics but suffer from a series of failures that reduce battery life increasing the cost of the system. Li-ion batteries solve the lead acid battery problems but the battery life cycle is affected by the drop in voltage that occurs during high discharge currents. The combination of a supercapacitor and a battery to form a HESS reduces the stress on the battery caused by the high discharge currents resulting in an increase in the life of the batteries. In this scenario, the main objective of the paper is to design and develop a photovoltaic converter with the addition of a HESS with Li-ion batteries for mid-range nominal power applications. The aim is to find solutions to cover a wide range of very necessary applications for the development of the current electric sector, such as the integration of renewable energies, smart grids, smart houses and electric vehicles (EVs).

### 1.4. Innovative Contribution

The main contribution of the paper is to develop a photovoltaic inverter in the power range of residential and large scale photovoltaic systems with the possibility of managing the power injection, in spite of being a renewable energy, by means of an energy storage system. As the limited lifetime of batteries represents a serious disadvantage, a hybrid combination of batteries and supercapacitors is implemented and studied for this power range. A method for sizing the energy storage system and the hybrid distribution based on the photovoltaic generation is also presented. The power sharing reduces the battery stress and increases its lifespan while providing constant power injection prior to grid necessities.

After the exhaustive literature review done in the previous section, the document is organized in the following manner: firstly, the topology is indicated together with the materials and methods developed for the experimental prototype. Secondly, the sizing of the ESS and the hybrid distribution based on sunny and cloudy day photovoltaic data is proposed. Then, the full control algorithm is presented, including as objectives the Maximum Power Point Tracking (MPPT) and reduced battery stress. Finally, the converter operation is validated via simulation and experimental results.

## 2. Topology

**Figure 2:**
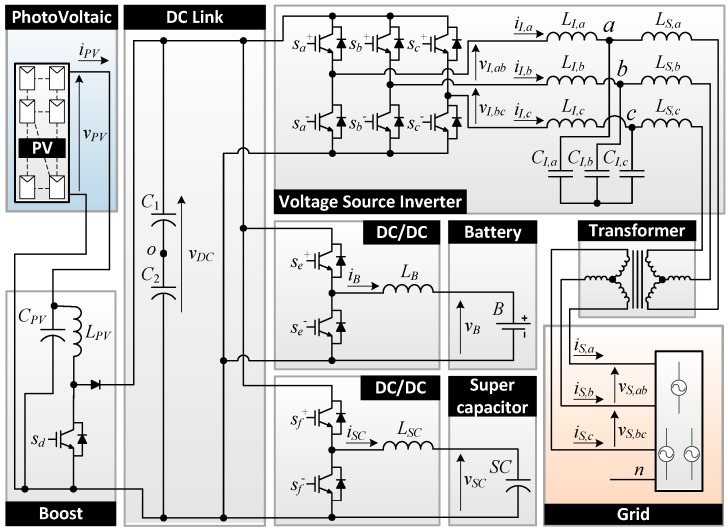
Topology of the photovoltaic inverter with hybrid energy storage system proposed.

The topology selected for the photovoltaic inverter with battery-supercapacitor HESS consists of four converters that share the DC link. It is composed by a boost stage for the PV source for solving MPPT, two bidirectional DC/DC converters for both ESS, the battery and the supercapacitor, for maintaining a DC link voltage, and a traditional 3-phase inverter for injecting a reference power into the grid. A transformer or DC current injection detection must be added for avoiding DC current injection into the grid. Figure 2 shows the scheme of the topology, which contains all the electrical variables used in subsequent sections.

## 3. Materials and Methods

**Figure 3:**
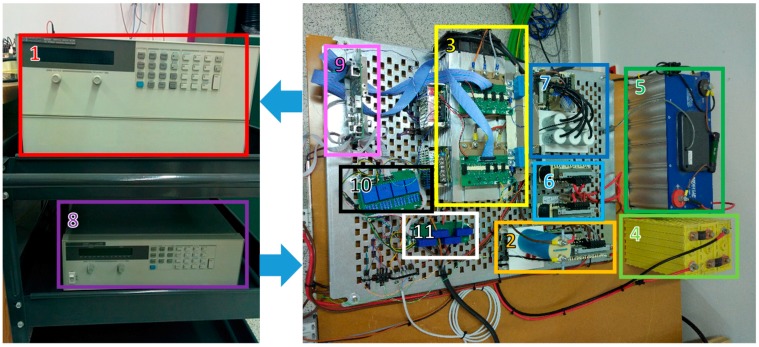
Picture of the prototype ((1) HP E4351B Solar Array Simulator, (2) Boost converter filter, (3) two Fuji IGBT-IPM 6MBP50RA120 power stage, (4) Li-ion batteries, (5) Maxwell BMOD0165 P048 B01 supercapacitor, (6) ESS converters filters, (7) inverter filter, (8) HP 6834B Power Source/Analyzer, (9) STM32F407 microcontroller based control board, (10) LV 25-P voltage sensors, (11) LA 55P/SP1 current sensors).

The proposed grid-connected PV HESS converter concept has been implemented experimentally to validate its operation. The experimental prototype is shown in Figure 3 and, basically, it contains the same elements as the topology shown in Figure 2. Each arrow in the diagram is a voltage or a current magnitude measured in the prototype by Hall Effect transducers, LV 25-P (LEM, Geneva, Switzerland) and LA 55P/SP1 (LEM, Geneva, Switzerland) respectively. The STM32F407 microcontroller-based control board acquires the measurements at a sample rate of 10 kHz, while the generated 5 kHz PWM signals to control the electronic switches are updated at the same rate, that is two times each period. The power semiconductor are two IGBT-IPM 6MBP50RA120 modules (Fuji Electric, Tokyo, Japan), where each of them includes three legs to implement the topology. MP 176065 Li-ion batteries (Saft, Bagnolet, France) and BMOD0165 P048 B01 supercapacitor (Maxwell Technologies, San Diego, CA, United States) are connected to the power stage by means of its filters. The PV array is emulated by the HP E4351B solar array simulator (Agilent Technologies, Santa Clara, California, United States) and the grid is emulated by the HP 6834B Power Source/Analyzer (Agilent Technologies, Santa Clara, CA, United States), both of them connected to the topology by the appropriate filter.

Voltage and current magnitudes are measured by Hall Effect transducers which provides galvanic isolation and both DC and AC components. For currents, the LA 55P/SP1 and for voltages the LV 25-P. Table 3 summarizes the most relevant specifications of both transducers. The main advantages of this type of sensors are the good linearity, low common mode and high precision however, they are usually more expensive than other alternatives.

**Figure 4:**
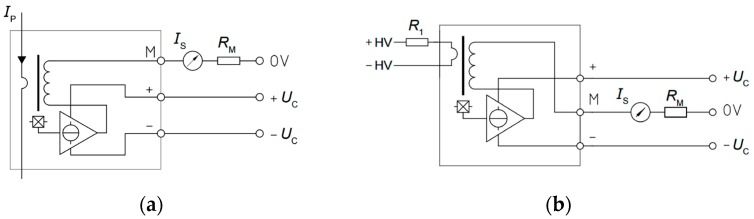
Hall effect transducers schematic: (**a**) LA 55P/SP1 current transducer; (**b**) LV 25-P voltage transducer.

LA 55P/SP1 current transducer schematic is shown in Figure 4a. Primary current *I_P_* travels through the ferromagnetic core and creates a magnetic flux, which is balanced by the magnetic flux created by the secondary current. An electronic stage is implemented in order to improve the accuracy of the primary current waveform representation by compensating the secondary current. By using this configuration, the bandwidth is extended and the time response is reduced.

The LV 25-P voltage transducer schematic is shown in Figure 4b. In this case, the measured voltage is turned to the primary current of the transducer by an external high value high power resistor *R*_1_. As the primary current will be quite small, a large internal primary coil with many turns is added with the aim of generate the proper primary magnetic flux. Except of that, the voltage transducer follows the same principle of operation the current transducer follows.

A Hall semiconductor connected to an error amplifier evaluate the net flow over the ferrite core. The amplifier output injects a current in the secondary winding in order to cancel the magnetic flux. Due to this fact, the Hall Effect closed-loop sensors are usually called ‘Zero-Flux’ sensor. Finally, the secondary current flows through an external resistor *R*_M_ for measuring a voltage drop that is proportional to the primary magnitude.

**Figure 5:**
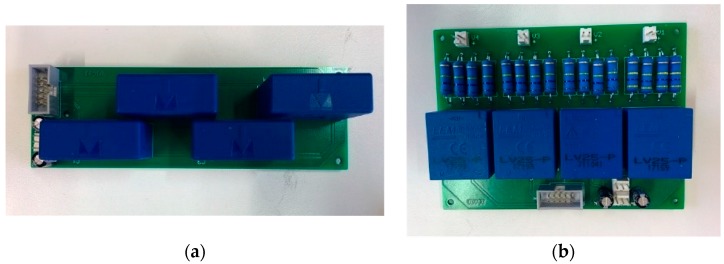
Sensors printed circuit boards for the experimental setup for measuring current and voltage magnitudes: (**a**) current sensors board and (**b**) voltage sensor board.

Four printed circuit board has been developed for the measuring the currents and voltages, two boards including four LA 55P/SP1 current transducers (see Figure 5a) and two boards including four LV 25-P voltage transducers (see Figure 5b).

## 4. HESS Sizing

The proper sizing of the ESS will meet the application needs while minimizing the cost, that is, the ESS capacity. The main goal of the ESS in a photovoltaic system is to turn a non-manageable power plant into a manageable one. One key factor when sizing the ESS is the time interval the inverter has to keep constant output active power. In Spain, some operation standards indicate that the power on the grid should be forecast every 15 min in order to supply the active and reactive power needed [36]. Also, the ESS should be operated inside a healthy SOC range which depends on the ESS technology, but in general it could be quantified as the 80% of the ESS rated capacity.

In this paper, the ESS sizing has been studied by simulating a constant power injection by the photovoltaic converter for 15 min intervals. These intervals are denominated as management time intervals. A range for the sizing will be proposed, the design value is a compromise between cost and photovoltaic discontinuities coverage.

**Figure 6:**
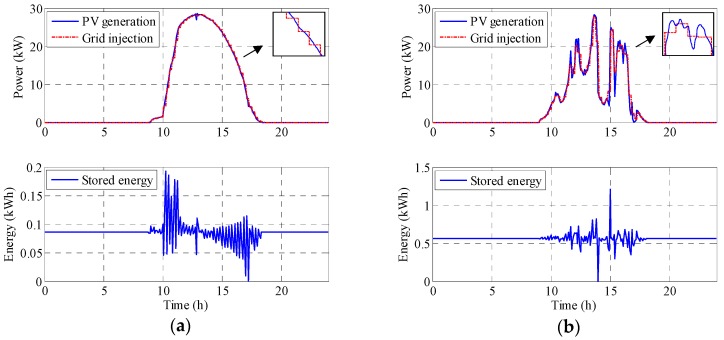
PV power generation, PV power injected into the grid (calculated as an average of the next 15 min interval forecast) and the energy stored: (**a**) for a sunny day and (**b**) for a cloudy day.

The minimum ESS capacity, $ESS_{Ca,min}$, is obtained by calculating the grid injected power as an average of the estimated power generation on the next management time interval. Obviously, this is an ideal management of the ESS, as it is based on the photovoltaic generation forecast. The mathematical expression in kWh applying the Tustin discrete approach is:

(1) $$ESS_{Ca,min}=\max{\left\{ESS_{Ch,i}+\left|\min{\left\{ESS_{Ch,i}\right\}}_{i=1}^{MN}\right|\right\}}_{i=1}^{MN}$$

(2) $$ESS_{Ch,i}=\overset{i}{\underset{k=0}{\sum }}\left(\frac{p_{S,k}+p_{S,k-1}-p_{PV,k}-p_{PV,k-1}}{2\times 3600}T_{s}\right)$$

(3) $$p_{S,i}=\overset{\left(m+1\right)N-1}{\underset{i=mN}{\sum }}\frac{p_{PV,i}}{N}\;\;\mathrm{for}\;mN<i<\left(m+1\right)N-1$$

where, *ESS_Ch_* is the energy charge in the ESS, *p_S_* is the grid injected power in kW, *p_PV_* is the photovoltaic generation power in kW, *T_s_* is sampling time in seconds, *i* is the sample pointer, *m* is the time management interval pointer, *k* is the cumulative pointer, *N* is the number of samples inside a management time interval and *M* is the number of management time intervals. Figure 6 shows the PV power generation, the PV power injected into the grid (constant in every 15 min interval and calculated as the average of the next management time interval) and the energy that must be stored. From this, it is possible to determine the minimum ESS size needed on a sunny and a cloudy day. Note that the asymmetry in the sunny day could be due to morning mist. The nominal sunny day generates 28 kW peak which implies a need for a minimum ESS of 0.2 kWh. However, the cloudy day has sharp changes and the ESS needed is close to 1.2 kWh.

The maximum ESS capacity, $ESS_{Ca,max}$, could be calculated as the energy needed to provide the nominal power of the photovoltaic inverter during a management time interval. In this way, the injected power reference could be matched in spite of a cloud covering over the PV panels during nominal photovoltaic generation at the beginning of the management time interval, assuring to provide the calculated power reference in the worst case. In kWh one has:

(4) $$ESS_{Ca,max}=\frac{p_{PV,nom}}{3600}T_{m}$$

where, *p_PV,nom_* is the nominal photovoltaic generation power in kW and *T_m_* is the management time interval in s. Considering a nominal PV power of 30 kW, the maximum ESS is 7.5 kWh.

Obviously, the higher the ESS size, the better from the management insurance point of view, but the cost and volume are also higher. The decreasing price of some battery technologies in the last years could make the maximum ESS size affordable for some installations.

In this work, the power reference for the next management time interval is calculated by considering the PV power at the end of the management time interval. With this technique and thinking of a sunny day, the ESS starts discharged at the beginning of the day, it charges during the first irradiance hours of the day, and finally, it discharges during the last irradiance hours of the day. This operation technique will improve the cycle and current rate charge/discharge of the battery, and therefore its lifetime. However, the ESS size is expected to be bigger than with the forecast technique.

**Figure 7:**
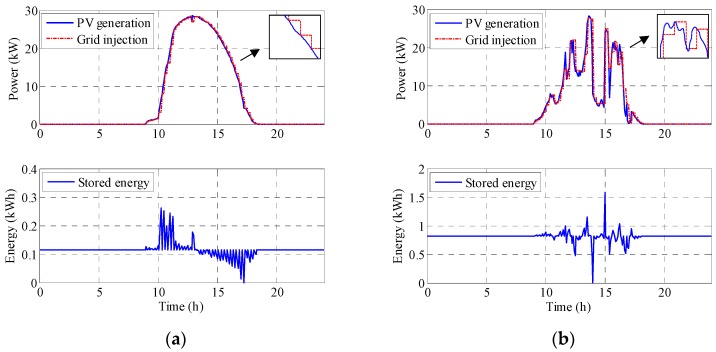
PV power generation, PV power injected into the grid (obtained from the PV power generation at the end of the previous 15-min interval) and the energy stored: (**a**) for a sunny day and (**b**) for a cloudy day.

Figure 7 shows the results obtained with the same 15 min cycle time. On a sunny day the required ESS is 0.26 kWh, while in a cloudy day it is 1.6 kWh. The expression is (1) again, but the grid injected power is obtained differently:

$$p_{S,i}=\frac{p_{PV,\left(m-1\right)N-1}}{3600N}\;\mathrm{for}\;mN<i<\left(m+1\right)N-1$$

For a battery-supercapacitor HESS, the calculated ESS size must be divided for assuring the energy density term for the battery and the power term for the supercapacitor, in order to maintain the stress of the battery and increase its lifetime even more. By using a first order LPF, the battery-supercapacitor energy percentage depends on the time constant τ, which is inversely proportional to the battery stress.

The HESS sizing criteria followed in this work is based on the percentage of energy each ESS provides when a step power reference is tracked. The photovoltaic generation changes over time, so taking into account a step power reference change is the worst scenario. The relation between *τ* and the management time interval gives the capacity percentage distribution for the battery, $ESS_{Ca,B}$, and for the supercapacitor, $ESS_{Ca,SC}$:

(6) ESSCa,B=(1−τTm)·ESSCa

(7) ESSCa,SC=τTm·ESSCa

where *ESS_Ca_* is the ESS capacity decided for the design. The value for the τTm ratio should be big enough in order to avoid sharp energy changes in the battery, which must be managed by the supercapacitor. Obviously, the compromise is located when the supercapacitor storage system becomes too expensive in the economic study of the converter. Considering the 15-min management time interval and the 7.5 kWh capacity of the ESS, a 0.1 index is a good balance as it implies the supercapacitor supports the battery for more than a minute assuring low battery stress [7] and the cost of the system is affordable [4].

## 5. Control Logic

**Figure 8:**
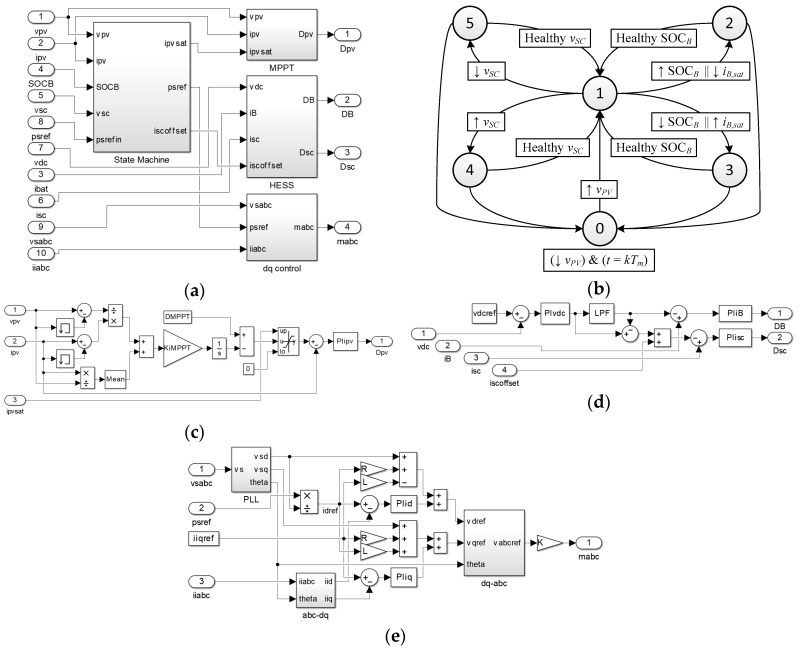
Control logic: (**a**) full scheme, (**b**) state machine, (**c**) MPPT, (**d**) HESS diagram and (**e**) *dq* diagram.

The control system scheme proposed in this work is shown in Figure 8a (readers may refer to Figure 2 to identify the variables). The measured electrical variables are placed on the left and the pulse width modulation (PWM) duty cycles control signals are placed on the right.

The higher level control is based on a state machine that generates the references to be tracked for the low level control blocks. These low level control blocks are the MPPT for the PV boost, the HESS for the battery-supercapacitor bidirectional DC/DC converters and the *dq* control for the grid connected inverter.

Figure 8b shows the state machine for controlling the grid connected photovoltaic inverter with battery-capacitor HESS. It is based on calculating the power reference to be injected by using the last PV power measures taken on the previous management time period, as explained before in Equation (5). The states are explained below:

1. The converter enters standby mode when the PV array has low irradiance, which could be evaluated via the PV voltage considering MPPT operation, and the management time interval starts.
2. In normal operation mode, every parameter of the converter is working inside the good operating range, and subsequently, the power injected into the grid is the reference power in the interval. This mode is reached from standby when the irradiance is enough, and from the other states when the ESS recovers a good SOC range.
3. The battery SOC has reached the top allowed level or the battery charging current becomes saturated. This means the PV generation is too high. In order to maintain the injected power reference, the system sets the reference power point tracking (RPPT) instead of the MPPT by saturating the PV boost current, *i_PV,sat_*. In case of high battery SOC, the RPPT is obtained for zero battery power flow and at the end of the management time interval, the PV boost stage operates on MPPT for the next *p_s,ref_* calculation in an optimistic way in order to force battery discharge. If the battery current is saturated, the RPPT is obtained to avoid current battery saturation.
4. Battery SOC is at the minimum value or battery current is saturated at its maximum value. This is a critical state, where *p_s,ref_* must change before the end of the management time interval for zero or non-saturated battery power flow because of low PV irradiance. *p_S,ref_* is calculated in a pessimistic way for the next cycle.
5. Supercapacitor has reached its top voltage. Negative power flow for the supercapacitor by adding a steady state negative offset, *i_SC,offset_*.
6. Supercapacitor voltage is too low. Positive power flow for the supercapacitor by adding a steady state positive offset.

The MPPT scheme is depicted in Figure 8c. It is based on the incremental conductance method [37]. The PV current reference is obtained by an integral controller or by limiting it via *i_PV,sat_* depending on the state. Then a PI controller tracks it by acting over the PV boost duty cycle.

The diagram in Figure 8d corresponds to the battery-supercapacitor control algorithm. The ESS is controlled with the aim of regulating the DC link voltage. First of all, a PI controller tracks the DC link voltage by calculating the ESS current reference. Then, the ESS current reference is divided into the HESS current reference by using a LPF. The battery current reference is the low frequency one, while the supercapacitor current reference is the high frequency one. Respective PI controllers adjust the duty cycle of the bidirectional DC-DC converters. In this way, the stress and temperature of the battery are reduced.

The inverter control diagram is shown in Figure 8e. It is based on the direct-quadrature theory, where the phase-neutral grid voltages, *v_abc_*, and currents, *i_abc_*, are transformed into the *dq* voltages, *v_dq_*, and currents, *i_dq_*, components for and easier current control [38]. The Phase Locked Loop (PLL) synchronizes with the fundamental positive sequence component of the grid voltage [39]. The *d* current reference is directly obtained by the grid power reference generated in the state machine. The *q* current reference is set to zero for null grid reactive power injection. Inverter reference voltages are obtained in the *dq* frame by using a feed forward scheme, which decouples *d* and *q* terms:

(8) $$v_{d,ref}=v_{d}-i_{d,ref}R+i_{q,ref}L+\frac{di_{d,ref}}{dt}L$$

(9) $$v_{q,ref}=v_{q}+i_{d,ref}L+i_{q,ref}R+\frac{di_{q,ref}}{dt}L$$

where, *L* is the grid filter inductor and *R* is the resistance in series. The derivative part is solved by using respective PI controllers and the inverter modulation index is calculated by scaling the *dq*-*abc* output depending on the DC link voltage.

## 6. Simulation

The proposed converter has been simulated with a MATLAB/Simulink Simscape model. The Simscape toolbox contains power electronics symbols that include semiconductor parameters for simulating switching losses, conduction losses and transients. Also, the simulation step for the power scheme was set to 10^−6^ s while the simulation step for the control algorithm was set to the sample rate the measurements are acquired (2 × 10^−4^ s). In this way, the simulation could be compared with the real behavior, as the power electronics are simulated with great detail and the control algorithm updates when the control board does.

The simulation test consists of 10 s where PV irradiance changes in all its range, and the time management interval is set to 2 s for checking the state machine operation. The parameters of the system are summarized in Table 4. The passive element of the topology has been designed in order to provide a low voltage and current ripple [40], while the rest of the parameters has been justified in previous sections.

**Figure 9:**
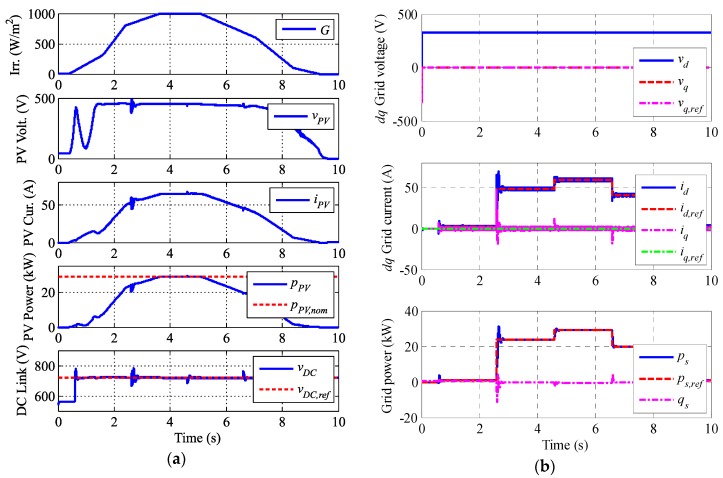

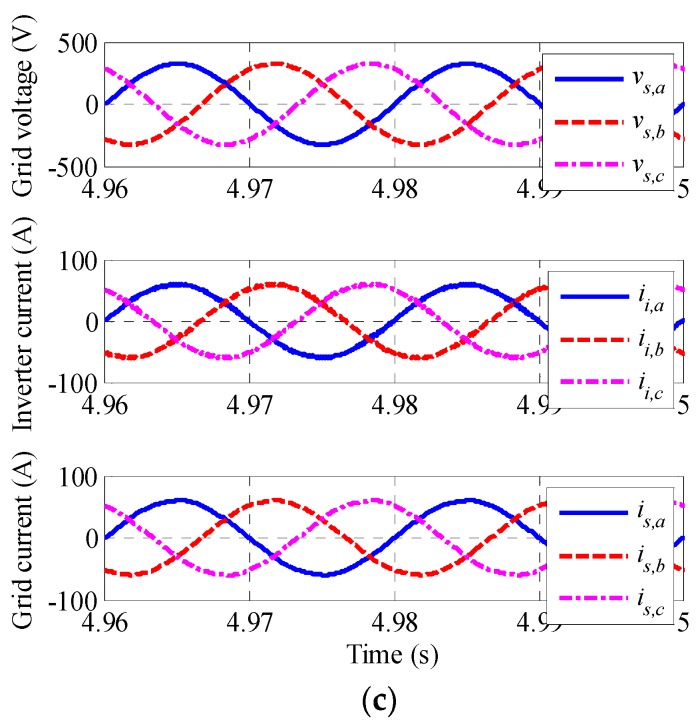
Simulation results. (**a**) Irradiance, PV output voltage; PV output current, PV output power and DC link voltage, (**b**) Voltage and current in the *dq* frame and power injected into the grid and (**c**) Grid voltage, inverter currents and grid currents.

**Figure 10:**
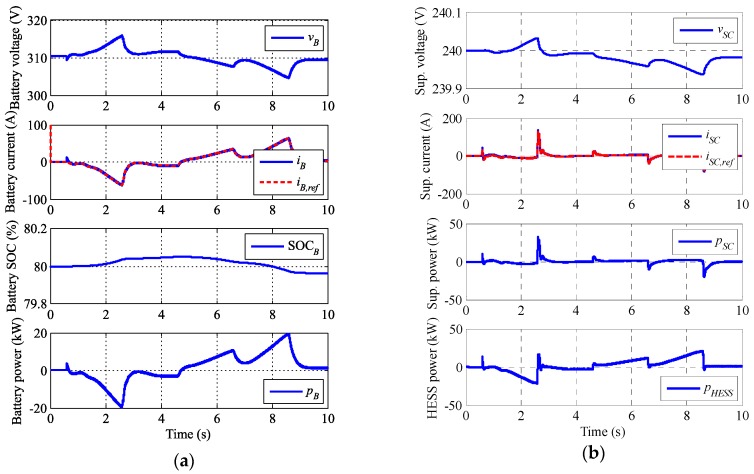
Simulation results: (**a**) Battery magnitudes and (**b**) supercapacitor magnitudes and HESS power.

Simulation results are shown in several figures. Figure 9a includes PV waveforms plus the DC link, Figure 9b,c include the grid variables, and Figure 10 includes the HESS response.

The sunny day profile can be seen in Figure 9a. The PV boost MPPT operates properly during the whole interval, except for the low irradiance startup where the power fluctuates a little. The HESS bidirectional DC/DC converters maintain the DC link voltage into the reference value.

Figure 9b represents the grid *dq* components and the injected power. The *d* voltage becomes constant while the *q* voltage is null which implies the PLL works properly. The injected power into then grid is totally active and constant during the management time intervals, making the PV plant manageable despite the irradiance changes. Figure 9c shows a window of the inverter and grid currents waveforms with nominal power injection. The total harmonic distortion (THD) obtained is less than the 5% the standards propose [41,42].

The battery and the supercapacitor operation are depicted in Figure 10a,b, respectively. Also, the total HESS operation is in Figure 10b. As expected, the battery shows a smooth operation with charge/discharge sequence, which means the battery stress and temperature is minimized and its lifetime is improved. The supercapacitor takes the sharp changes of voltage. Battery and supercapacitor packs are associated in order to decrease the boost index to the DC link, where the performance and controllability is better. The simulations stay away from battery and supercapacitor SOC and saturation limits, so they do not affect in the test.

## 7. Experimental Results

**Figure 11:**
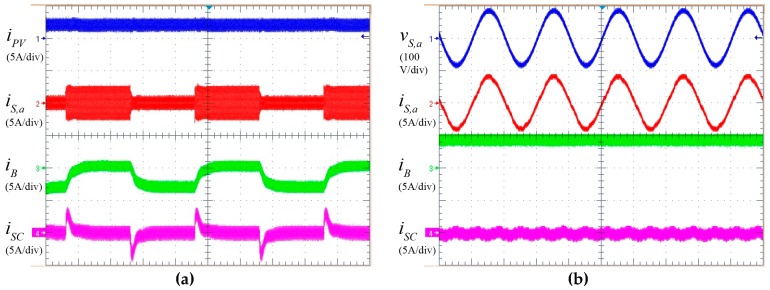
Experimental results. (**a**) PV current, grid current, battery and supercapacitor current for a cyclic injected power reference operation. (**b**) Grid voltage, grid current, battery and supercapacitor current for steady state operation.

Experimental results are shown in Figure 11. Figure 11a depicts a cyclic injected power reference operation mode in order to demonstrate how the HESS works while the batteries are charging. In that case, the PV maintains its power injection in the MPPT while the grid power reference changes as a square wave and the battery together with the supercapacitor compensate the power energy balance. Note how the sharp energy changes in the ESS are absorbed by the supercapacitor maintaining the battery in a healthier operation mode.

Figure 11b depicts a steady state operation mode with the aim of checking the grid waveforms while the battery is discharging. The power factor is one as both the voltage and the current are in phase and the current harmonic distortion stays in low terms. In the steady state only the battery is the one that provides energy in the ESS. The experimental behavior matches the expected behavior described in the simulation section.

## 8. Conclusions

A grid-connected photovoltaic inverter with battery-supercapacitor HESS for providing manageable power injection has been presented. An adapted combination of converter topologies has been selected. The system components were designed in order to match the required behavior, taking into account different irradiance conditions based on a typical daily profile. The control logic was implemented with the objectives of: (1) extracting the maximum power from the PV panels (MPPT control), (2) suitable task sharing between battery and SC and (3) injecting power into the grid based on the direct-quadrature theory.

The main benefit of the proposed system is the possibility to transform a PV plant in a manageable power plant. This is due to the constant power injection to the grid during 15-min intervals, which is a value often considered by several standards. In addition, a fraction of the battery current is diverted to the SC along with the high frequency components. Because of this, there is a significant reduction in charge/discharge current rates, which leads to a longer lifetime and permits a reduction in size of the battery.

The proposed topology and strategy are experimentally validated for sudden change in the reference power to inject into grid. The results show that the proposed scheme is able to: (a) reduce the charge/discharge rates and (b) produce a nearly sinusoidal waveform for the current injected into the grid. Also, the sizing of the ESS and the battery/supercapacitor distribution has been addressed by using the power curves of the PV array. A range is calculated for the capacity of the ESS depending on the nominal power of the converter and the time interval the generation wants to be manageable.

## Figures and Tables

**Table 1:** Summary of HESS smaller than 5 kW in the literature.

Paper	[9]	[10]	[11]	[12]	[13]	[14]	[15]	[16]	[17]
**Application**	Pulse-operated power systems	Load supply	Energy harvesting	Load supply	Load supply	Load supply	Load supply	Load supply	Load supply
**System**	DC	DC	DC	DC	DC	DC + 3~Alternating Current (AC)	DC	DC	DC
**Topology**	Active Hybrid Bidirectional DCDC	Bidirectional DCDC	PV Boost + Bidirectional DCDC	Bidirectional DCDC	PV Boost Bidirectional DCDC	PV Boost + Bidirectional DCDC + 3~Inverter	PV Boost + Bidirectional DCDC	Bidirectional DCDC	PV Boost + Bidirectional DCDC
**Rated Power**	132 W	500 W	5 W	50 W	100 W	1 kW	50 W	100 W	2 kW
**Comparison**	Passive vs. Active hybrid	Napoli vs. MIAD	-	-	-	LPF vs. Haar wavelet vs. Fuzzy	-	-	ESS vs. HESS
**Sizing**	-	-	Statistical analysis	-	-	-	Optimization flowchart	-	-
**Control**	Proportional Integral (PI)	Multiplicative-increase-additive-decrease (MIAD)	State machine	Predictive control	PI with LPF	Multimode fuzzy-logic power allocator	-	state-space averaged model	Flatness approach
**Goals**	More power Lower battery temperature Longer battery lifetime	Minimization of the battery current fluctuation and SC energy loss	Increase battery lifetime	Battery current and state of charge SC into the limits	Improve the life span and reduce the current stresses on battery	Avoid depleting or saturating the two components Relaxing the stress on batteries	Healthy SOC	Control design	Role of SC as a transient power source

**Table 2:** Summary of HESS greater than 5 kW in the literature.

Paper	[18]	[19]	[20]	[21]	[22,23]	[24]	[25]	[26]	[27]
**Application**	Microgrids	UPS	Hybrid microgrid	Remote Area Power Supply	Grid-connected photovoltaic	Microgrid	Microgrid	Microgrid	Power sharing
**System**	DC	DC	DC + 3~AC		DC + 3~AC	DC + 3~AC	DC + 3~AC	DC + 3~AC	DC + 3~AC
**Topology**	Dual active bridge	Bidirectional DCDC	PV Boost + 3~Inverter and Bidirectional DCDC + 3~Inverter	Wind Rectifier-Boost + Bidirectional DCDC + 3~Inverter	PV Boost + Bidirectional DCDC + 3~Inverter	Bidirectional DCDC + 3~Inverter	PV Boost + 3~Inverter and Bidirectional DCDC + 3~Inverter	PV Boost + Bidirectional DCDC + 3~Inverter	Bidirectional DCDC + 3~Inverter
**Rated Power**	5 kW Modular	500 kVA	30 kW	25 kW	1 MW	10 kW	60 kW	100 kW	4 MW
**Comparison**	-	-	Different capacities	LPF	-	two-loop PI control and PI sliding mode	-	-	Multi-objective optimization problem (MOP), LPF and LUT
**Sizing**	-	Backup time	Power grading	Equations	SC as 1/5 battery	-	Monte Carlo capacity model	-	-
**Control**	LPF Energy management	Power sharing	State of charge with load control	Energy management algorithm	Semischeduled generation	Sliding mode	Hysteretic loop	PI controllers and Direct Power Control	Linear weighted summation algorithm
**Goals**	Energy balance with renewables	Optimal SC-battery combination vs SC cost	Power balance and ESS in healthy state	Robust voltage and frequency regulation Effective HESS management	Avoid low power level battery operation	Using a nonlinear controller	Extend the battery lifetime by avoiding small cycles	Maintain the grid power demand coming from the grid operator	Optimization of the energy loss and state of charge of the SC

**Table 3:** Characteristics of AC and DC voltage and current sensors.

Current Transducers	Specification	Voltage Transducers
0 ... ±100 A	Measuring range	0 ... ±14 mA (10 mA/500 V)
25 mA	Secondary nominal current rms	25 mA
±12 ... 15 V	Supply voltage	±15 V (±5%)
200 kHz	Frequency bandwith	200 kHz
2.5 kV rms	Isolation	2.5 kV rms
1:2000	Conversion ratio	2500:1000
±0.9%	Accuracy	±0.9%
−40 °C to 85 °C	Operating temperature	0 °C to 70 °C

**Table 4:** Simulation parameters.

Symbol	Value	Symbol	Value	Symbol	Value
*p_PV,nom_*	30 kW	*ESS_Ca,B_*	3 kWh	*L_PV_*	10 mH
*T_m_*	2 s	*ESS_Ca,SC_*	0.3 kWh	*L_B_ = L_SC_*	20 mH
*τ*	0.2 s	*PWM*	5 kHz	*LCL*	5 mH, 10 μF, 5 mH
*v_DC,ref_*	720 V	*C*_1_*, C*_2_	2.2 mF	*C_SC_*	37.5 F

## Acronyms

AC	Alternating Current
B	Battery
CAES	Compressed Air Energy Storage
DC	Direct Current
DER	Distributed Energy Resource
*dq*	Direct-quadrature
ESS	Energy Storage Systems
HESS	Hybrid Energy Storage Systems
LPF	Low Pass Filter
MPPT	Maximum Power Point Tracking
PHS	Pumped Hydropower Storage
PI	Proportional Integral
PLL	Phase Locked Loop
PV	PhotoVoltaic
PWM	Pulse Width Modulation
RPPT	Reference Power Point Tracking
SC	SuperCapacitors
SOC	State Of Charge
THD	Total Harmonic Distortion
UPS	Uninterruptible Power Supply

## Nomenclature

a, b, c	Grid phases
$C_{I,a}$; $C_{I,b}$; $C_{I,c}$	Inverter output filter capacitors
$C_{1}$, $C_{2}$	DC link capacitors
$C_{PV}$	PV boost stage capacitor
$D_{B}$	DC/DC battery converter duty cycle
$D_{PV}$	Boost converter duty cycle
$D_{SC}$	DC/DC supercapacitor converter duty cycle
$ESS_{Ca,B}$	Battery ESS capacity
$ESS_{Ca,max}$	Maximum ESS capacity needed for a certain design
$ESS_{Ca,min}$	Minimum ESS capacity required for a certain design
$ESS_{Ca,SC}$	Supercapacitor ESS capacity
$ESS_{Ca}$	Full ESS capacity
$ESS_{Ch}$	Energy charge in the ESS
i	sample pointer
$i_{d,ref}$	Grid direct reference current
$i_{I,a}$; $i_{I,b}$; $i_{I,c}$	Inverter output currents
$i_{PV,sat}$	PV current saturation for RPPT
$i_{q,ref}$	Grid quadrature reference current
$i_{S,a}$; $i_{S,b}$; $i_{S,c}$	Grid currents
$i_{SC,offset}$	Offset current term for the supercapacitor converter
$i_{B}$	Battery current
$i_{dq}$	*Grid dq* current
$i_{PV}$,	PV module current
$i_{SC}$	Supercapacitor converter current
k	cumulative pointer
L	Equivalent grid filter indtor
$L_{I,a}$; $L_{I,b}$; $L_{I,c}$	Inverter output filter inductances (inverter side)
$L_{S,a}$; $L_{S,b}$; $L_{S,c}$	Inverter output filter inductances (grid side)
$L_{B}$	DC/DC battery converter inductance
$L_{PV}$	Boost converter inductance
$L_{S}$	Inverter output filter inductances (grid side)
$L_{SC}$	DC/DC supercapacitor converter inductance
M	number of management time intervals
m	time management interval pointer
$m_{abc}$	Inverter duty cycle
N	number of samples inside a management time interval
o	Middle point of the DC link
$p_{PV,nom}$	Nminal power of the converter
$p_{s,\mathrm{ref}}$	Reference active power set-point
$p_{B}$	Battery power
$p_{PV}$	Photovoltaic generation power
$p_{PV}$	PV module power
$p_{S}$	Grid injected power
$p_{SC}$	Super-capacitor power
R	Equivalent grid filter resistance
$R_{M}$	External measurement resistance for LEM transducers
$S_{a}^{+}$; $S_{b}^{+}$; $S_{c}^{+}$; $S_{a}^{-}$; $S_{b}^{-}$; $S_{c}^{-}$	Inverter switching signals
$S_{d}$	Boost converter switching signal
$S_{e}^{+}$; $S_{e}^{-}$	DC/DC battery converter switching signals
$S_{f}^{+}$; $S_{f}^{-}$	DC/DC supercapacitor converter switching signals
$T_{m}$	Management time interval
$T_{s}$	Sampling time
$v_{d,ref}$	Grid direct reference voltage
$v_{DC,\mathrm{ref}}$	DC link voltage reference value
$v_{I,\;a}$; $v_{I,\;b};\;v_{I,\;c}$	Inverter output voltages
$v_{q,ref}$	Grid quadrature reference voltage
$v_{S,\;a}$; $v_{S,\;b};\;v_{S,\;c}$	Grid Voltages
$v_{B}$	Battery voltage
$v_{d}$	Grid direct voltage
$v_{DC}$	DC link voltage
$v_{dq}$	*Grid dq* voltages
$v_{PV}$	PV module voltage
$v_{q}$	Grid quadrature voltage
$v_{SC}$	Supercapacitor voltage
τ	Low-pass filter time constant

## References

[1] Chen H., Cong T., Yang W., Tan C., Li Y., Ding Y. (2009). Progress in electrical energy storage system: A critical review. Prog. Natl. Sci..

[2] Ippolito M.G., Telaretti E., Zizzo G., Graditi G. A new device for the control and the connection to the grid of combined RES-based generators and electric storage systems. Proceedings of the 4th International Conference on Clean Electrical Power: Renewable Energy Resources Impact, ICCEP 2013.

[3] Xu L., Ruan X., Mao C., Zhang B., Luo Y. (2013). An Improved Optimal Sizing Method for Wind-Solar-Battery Hybrid Power System. IEEE Trans. Sustain. Energy.

[4] Zakeri B., Syri S. (2015). Electrical energy storage systems: A comparative life cycle cost analysis. Renew. Sustain. Energy Rev..

[5] Srivastava A., Kumar A., Schulz N. (2012). Impact of Distributed Generations with Energy Storage Devices on the Electric Grid. IEEE Syst. J..

[6] Meshram S., Agnihotri G., Gupta S. (2013). Performance Analysis of Grid Integrated Hydro and Solar Based Hybrid Systems. Adv. Power Electron..

[7] Kuperman A., Aharon I. (2011). Battery–ultracapacitor hybrids for pulsed current loads: A review. Renew. Sustain. Energy Rev..

[8] Guerrero-Martínez M.A., Romero-Cadaval E., González F.B., Montero M.I.M., Romera E.G. (2009). Supercapacitors: Alternative Energy Storage Systems. Przegląd Elektrotechniczny.

[9] Gao L., Dougal R., Liu S. (2005). Power enhancement of an actively controlled battery/ultracapacitor hybrid. IEEE Trans. Power Electr..

[10] Choi M., Kim S., Seo S. (2012). Energy Management Optimization in a Battery/Supercapacitor Hybrid Energy Storage System. IEEE Trans. Smart Grid.

[11] Ongaro F., Saggini S., Mattavelli P. (2012). Li-Ion Battery-Supercapacitor Hybrid Storage System for a Long Lifetime, Photovoltaic-Based Wireless Sensor Network. IEEE Transac. Power Electr..

[12] Hredzak B., Agelidis V., Jang M. (2014). A Model Predictive Control System for a Hybrid Battery-Ultracapacitor Power Source. IEEE Trans. Power Electr..

[13] Kollimalla S., Mishra M., Narasamma N. (2014). Design and Analysis of Novel Control Strategy for Battery and Supercapacitor Storage System. IEEE Trans. Sustain. Energy.

[14] Feng X., Gooi H., Chen S. (2014). Hybrid Energy Storage with Multimode Fuzzy Power Allocator for PV Systems. IEEE Trans. Sustain. Energy.

[15] Glavin M., Hurley W. (2012). Optimisation of a photovoltaic battery ultracapacitor hybrid energy storage system. Sol. Energy.

[16] Jung H., Wang H., Hu T. (2014). Control design for robust tracking and smooth transition in power systems with battery/supercapacitor hybrid energy storage devices. J. Power Source.

[17] Benaouadj M., Aboubou A., Ayad M., Becherif M. (2014). Nonlinear Flatness Control Applied to Supercapacitors Contribution in Hybrid Power Systems Using Photovoltaic Source and Batteries. Energy Procedia.

[18] Zhou H., Bhattacharya T., Tran D., Siew T., Khambadkone A. (2011). Composite Energy Storage System Involving Battery and Ultracapacitor with Dynamic Energy Management in Microgrid Applications. IEEE Trans. Power Electr..

[19] Lahyani A., Venet P., Guermazi A., Troudi A. (2013). Battery/Supercapacitors Combination in Uninterruptible Power Supply (UPS). IEEE Trans. Power Electr..

[20] Zhu Y., Zhuo F., Shi H. Power management strategy research for a photovoltaic-hybrid energy storage system. Proceedings of the 2013 IEEE ECCE Asia Downunder.

[21] Mendis N., Muttaqi K., Perera S. (2014). Management of Battery-Supercapacitor Hybrid Energy Storage and Synchronous Condenser for Isolated Operation of PMSG Based Variable-Speed Wind Turbine Generating Systems. IEEE Trans. Smart Grid.

[22] Wang G., Ciobotaru M., Agelidis V. (2014). Power Smoothing of Large Solar PV Plant Using Hybrid Energy Storage. IEEE Trans. Sustain. Energy.

[23] Wang G., Ciobotaru M., Agelidis V. (2016). Power Management for Improved Dispatch of Utility-Scale PV Plants. IEEE Trans. Power Syst..

[24] Etxeberria A., Vechiu I., Camblong H., Vinassa J. (2011). Comparison of Sliding Mode and PI Control of a Hybrid Energy Storage System in a Microgrid Application. Energy Procedia.

[25] Jia H., Mu Y., Qi Y. (2014). A statistical model to determine the capacity of battery–supercapacitor hybrid energy storage system in autonomous microgrid. Int. J. Electr. Power Energy Syst..

[26] Choudar A., Boukhetala D., Barkat S., Brucker J. (2015). A local energy management of a hybrid PV-storage based distributed generation for microgrids. Energy Conver. Manag..

[27] Jiang W., Zhang L., Zhao H., Huang H., Hu R. (2016). Research on power sharing strategy of hybrid energy storage system in photovoltaic power station based on multi-objective optimization. IET Renew. Power Gener..

[28] Chatterjee S., Pandey K.G. (2003). Thermoelectric cold-chain chests for storing/transporting vaccines in remote regions. Appl. Energy.

[29] Muñoz-García M.A., Moreda G.P., Raga-Arroyo M.P., Marín-González O. (2013). Water harvesting for young trees using Peltier modules powered by photovoltaic solar energy. Comput. Electron. Agric..

[30] Spertino F., Graditi G. (2014). Power Conditioning Units in Grid-Connected Photovoltaic Systems: a Comparison with Different Technologies and Wide Range of Power Ratings. Sol. Energy.

[31] Nema S., Nema R.K., Agnihotri G. (2011). Inverter Topologies and Control Structure in Photovoltaic Applications: A Review. J. Renew. Sustain. Energy.

[32] Salas V., Olías E. (2011). Overview of the State of Technique for PV Inverters used in Low Voltage Grid-Connected PV Systems: Inverters above 10 kW. Renew. Sustain. Energy Rev..

[33] Romero-Cadaval E., Spagnuolo G., Franquelo L.G., Ramos-Paja C., Suntio T., Xiao W. (2013). Grid-Connected Photovoltaic Generation Plants: Components and Operation. IEEE Ind. Electr. Mag..

[34] Khaligh A., Li Z. (2010). Battery, Ultracapacitor, Fuel Cell, and Hybrid Energy Storage Systems for Electric, Hybrid Electric, Fuel Cell, and Plug-In Hybrid Electric Vehicles: State of the Art. IEEE Trans. Veh. Technol..

[35] Adinolfi G., Graditi G., Siano P., Piccolo A. (2015). Multi-Objective Optimal Design of Photovoltaic Synchronous Boost Converters Assessing Efficiency, Reliability and Cost Savings. IEEE Transac. Ind. Inf..

[36] P.O. 3.1 Programación de la Generación. http://www.boe.es/boe/dias/2015/12/19/pdfs/BOE-A-2015-13875.pdf.

[37] Esram T., Chapman P.L. (2007). Comparison of Photovoltaic Array Maximum Power Point Tracking Techniques. IEEE Trans. Energy Conver..

[38] Roncero-Clemente C., Romero-Cadaval E., Roncero-Sanchez P., Gonzalez-Romera E. Comparison of two power flow control strategies for photovoltaic inverters. Proceedings of the 38th Annual Conference on IEEE Industrial Electronics Society (IECON).

[39] Miñambres V., Milanés M.I., Vinagre B., Romero E. (2009). Phase Locked Loop for Distorted Three-Phase Systems tested with a PI, a PID and a Fractional PI. Przegląd Elektrotechniczny.

[40] Hamza K.E., Linda H., Cherif L. LCL filter design with passive damping for photovoltaic grid connected systems. Proceedings of the 6th International Renewable Energy Congress (IREC).

[41] IEEE (2003). IEEE Std 1547: IEEE Standard for Interconnecting Distributed Resources With the Electric Power System.

[42] IEC (2001). IEC 61727 ed2.0: Photovoltaic (PV) Systems–Characteristics of the Utility Interface.

